# Prolonged KI Polyomavirus Infection in Immunodeficient Child

**DOI:** 10.3201/eid1804.111588

**Published:** 2012-04

**Authors:** Valeria Falcone, Marcus Panning, Brigitte Strahm, Thomas Vraetz, Sibylle Bierbaum, Dieter Neumann-Haefelin, Daniela Huzly

**Affiliations:** Freiburg University Medical Center, Freiburg, Germany

**Keywords:** KIPyV, WUPyV, cytomegalovirus, CMV, EBV, BKV, BKPyV, DNA, immunocompromised, polyomavirus, coinfection, hematopoietic stem cell transplant, HSCT, human bocavirus, viruses

**To the Editor:** Two novel polyomaviruses (PyVs), KIPyV and WUPyV, were identified in respiratory and fecal specimens from children with signs and symptoms of respiratory tract infection (*1**,**2*). A review of literature on emerging viruses in transplant recipients indicated that up to 80% of patients harboring these PyVs are co-infected with another respiratory virus, complicating interpretation of positive findings (*3*). Seroprevalence of KIPyV and WUPyV in healthy blood donors in Germany have been reported to be 67% and 89%, respectively (*4*).

The effect of these viruses in immunocompromised patients is unknown. Some studies report a higher frequency of KIPyV DNA detection in hematopoietic stem cell transplant (HSCT) recipients (*5**–**7*) than in immunocompetent patients. In fact, HCST recipients might be more prone to productive infection with KIPyV and WUPyV than to infection with PyVs JC and BK (BKPyV) (*5*).

We report prolonged detection of KIPyV DNA in the respiratory tract of an immunocompromised child. A 12-year-old girl with severe combined immunodeficiency was admitted to the Freiburg University Medical Center, Germany, in November 2009 for treatment of progressive respiratory problems and cytomegalovirus (CMV) disease. Although the molecular basis of the immune disorder was unknown, HSCT was indicated because of uncontrolled CMV infection and progressive clinical deterioration.

Allogenic HSCT was performed in February 2010. Pretransplant treatments included thyothepa (day −7; 8 mg/kg), fludarabine (days −6 to −3; 120 mg/m^2^), treosulfan (days –6 to – 4; 42 g/m^2^), and antithymocyte globulin (days −4 to −2; 45 mg/kg). The patient received bone marrow cells (4.2 × 10^6^ CD34-positive cells/kg) from an 8/10 human leukocyte antigen-matched, CMV-positive, unrelated donor. Graft-versus-host disease prophylaxis consisted of cyclosporine A (from day −1) and methotrexate (days +1, +3, +6; 10 mg/m^2^). Leukocyte, granulocyte, and platelet engraftment occurred on days +18, +19, and +32, respectively. Full donor chimera was detected by day +62 (Figure, panel A).

**Figure:**
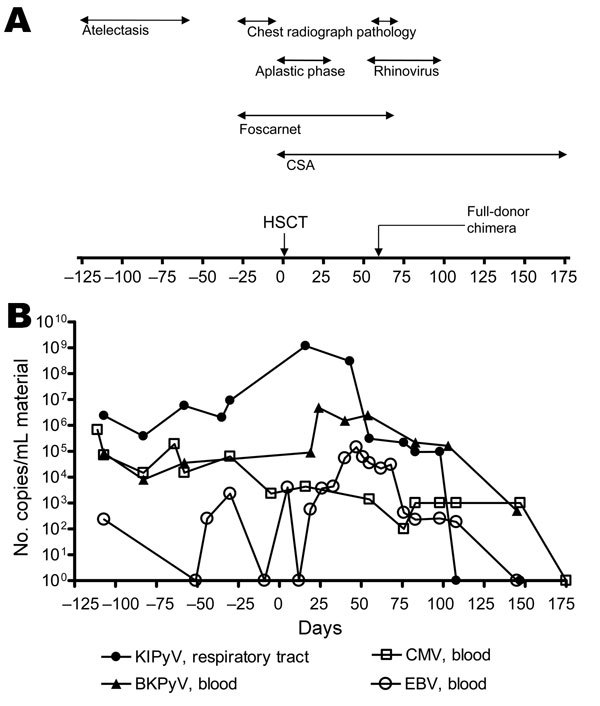
Timeline of clinical and virologic features for a 12-year-old immunocompromised child, before and after HSCT, Germany, 2009. A) Main clinical events and therapeutic measures. B) Viral DNA load measured by real-time PCR. CSA, cyclosporin A; HSCT, hematopoietic stem cell transplant; KIPyV, KI polyomavirus; BKPyV, BK polyomavirus; CMV, cytomegalovirus; EBV, Epstein-Barr virus.

Before hospitalization, the child had had several pulmonary infections. At admission, chest radiograph showed middle lobe atelectasis but no visible infiltrates. On day −83, human bocavirus was detected. On day −27, the occurrence of bilateral infiltrates was assessed, and pneumonia was diagnosed. On day +55, fever and hypoxia were monitored; chest radiograph revealed regressive infiltrates in the lower lobes but central infiltrates in the upper lobes. Rhinovirus RNA was detected at this time and persisted in the respiratory tract until day +98 (Figure, panel A). Retrospectively, KIPyV DNA was detected in 6 nasopharyngeal aspirate specimens, 4 throat swab specimens, and 1 bronchoalveolar lavage specimen collected between days −103 and +98 (Figure, panel B). No KIPyV was detected in EDTA-treated blood samples at any time. Stool samples were not available. The highest level of KIPyV DNA (10^9^ copies/mL) was detected on day +16. Starting from day +43, a steady decrease in KIPyV viral load was observed. Phenotypical analysis of blood leukocytes on day +55 showed normal CD56+/16+ natural killer cells and good T-cell engraftment but no B cells. On day +108, viral clearance had occurred. Sequencing of the small t antigen amplified from all available samples was performed (*8*) and showed 100% nucleotide identity (GenBank accession no. JN874415).

A central indication for performing HSCT was uncontrolled CMV infection. Before transplantation, while the patient was receiving gancyclovir treatment, she had a high CMV DNA load (Figure, panel B). A typical mutation in the UL97 gene, conferring resistance to gancyclovir, was confirmed. Antiviral therapy was switched to foscarnet (day −30 to day +70), and CMV DNA drastically decreased. After T-cell engraftment, further decrease was followed by decrease of Epstein-Barr virus and BKPyV DNA load, which had considerably increased until day +50 (Figure, panel B).

In this immunocompromised child, KIPyV DNA was in the respiratory tract for 7 months. High prevalence of KIPyV in HSCT patients has been reported, suggesting that T-cell impairment might be a factor in facilitating KIPyV replication (*5**,**7*). Cellular immunity is crucial for containing CMV and BKPyV replication (*9*). Our observations also support a central role for cell-mediated immunity in controlling KIPyV. In fact, the peak of KIPyV DNA replication occurred during the aplastic phase. Moreover, a concomitant decrease in KIPyV, CMV, Epstein-Barr virus, and BKPyV DNA load observed after noting normal CD56+/16+ natural killer cells and T-cell engraftment further supports this hypothesis. Therefore, we theorize that early infection in childhood with KIPyV probably results in latency in the presence of a functional immune system. Immune impairment might result in reactivation. Sequence identity of KIPyV DNA from sequentially collected respiratory samples in this case further supports the conclusion that reactivation, rather than reinfection by heterologous strains, occurred.

The contribution of KIPyV to respiratory disease remains ambiguous: in the posttransplantation period, rhinovirus detection correlates with increasing pulmonary infiltrates. However, before transplantation, KIPyV was identified as the sole agent in the respiratory tract; increasing viral loads seem to correlate with the development of bilateral infiltrates. The relevance of human bocavirus co-detection in 1 sample before transplantation remains unclear. Future prospective studies are needed to establish a correlation between immunosuppression, KIPyV shedding, and the occurrence of respiratory symptoms in immunocompromised patients.
